# Gute Wiedererlangung von Freizeitaktivitäten und Sport nach Primärimplantation von zementfreien Knietotalendoprothesen nach 5 Jahren: Eine retrospektive Kohortenstudie

**DOI:** 10.1007/s00132-025-04717-5

**Published:** 2025-09-17

**Authors:** Manish Theiner, Julian Mehl, Norbert Freund

**Affiliations:** 1 Krankenhaus der Barmherzigen Schwestern Ried, 4910 Ried im Innkreis, Österreich; 2 Klinikum der Technischen Universität München (TUM Klinikum), München, Deutschland; 3 UWK Donau Krems, Krems, Österreich

**Keywords:** Freizeitaktivitäten, Zementfrei, Patient reported outcome measures, Return to Sport, Totaler Kniegelenkersatz, Leisure activities, Cementless, Patient reported outcome measures, Return to sport, Total knee replacement

## Abstract

**Einleitung:**

Die Prognose der Wiederaufnahme sportlicher Aktivitäten nach einer Knie-Totalendoprothese (K-TEP) ist für viele Patienten entscheidend. Obwohl zementfreie Implantationstechniken zunehmend Anwendung finden, fehlen belastbare Langzeitdaten zu deren Einfluss auf sportliche Betätigung. Ziel dieser Studie war die Analyse individueller Freizeitaktivitäten sowie sportlicher und funktioneller Parameter nach zementfreier K-TEP über einen Zeitraum von fünf Jahren.

**Material und Methoden:**

In einer retrospektiven Analyse wurden prospektiv erhobene Daten von 42 Patienten (Alter 35–80 Jahre, Implantat: Attune, DePuy) ausgewertet. Für jeden Patienten wurden die drei relevantesten Sportarten anhand des Knee Society Scores (KSS) erhoben und prä- mit postoperativen Werten (nach 1 und 5 Jahren) verglichen. Zusätzlich wurden Subskalen des KSS und des Knee Injury and Osteoarthritis Outcome Score (KOOS) analysiert. Die statistische Auswertung erfolgte mittels Wilcoxon-, Friedman- und Spearman-Test.

**Ergebnisse:**

Von den 42 eingeschlossenen Patienten waren 73,8 % weiblich. Der Altersdurchschnitt lag bei 64,02 ± 9,34 Jahren. Im Vergleich zu den präoperativen Werten zeigten die PROM eine signifikante Verbesserung der sportlichen Leistungsfähigkeit (*p* < 005) sowohl nach 1 als auch nach 5 Jahren. Nach 5 Jahren zeigte sich die Verbesserung wie folgt: Bei den Lieblingssportarten zeigte sich eine Reduktion der Beschwerden von „stark“ auf „wenig“. Die KSS-Sportuntergruppen zeigten eine Reduktion von „sehr stark-stark“ zu „mittelmäßig-wenig“. Die KOOS-Sportuntergruppen zeigten eine Reduktion der Beschwerden von „extrem-stark“ zu „moderat“. Die Korrelationsuntersuchung der beiden Sportuntergruppen zeigten eine hohe Vergleichbarkeit. Es traten keine aseptischen Lockerungen oder periprothetischen Infektionen auf.

**Diskussion:**

Die PROMs zeigten signifikante Verbesserungen der sportlichen Leistungsfähigkeit (*p* 0,05) nach 1 und 5 Jahren. Die Beschwerden bei den Lieblingssportarten reduzierten sich von „stark“ auf „wenig“. Die KSS-Sportuntergruppen zeigten eine Reduktion von „sehr stark–stark“ zu „mittelmäßig–wenig“, während sich die KOOS-Sportuntergruppen von „extrem–stark“ zu „moderat“ verbesserten. Die beiden Scores zeigten eine hohe Korrelation. Es traten keine aseptischen Lockerungen oder Infektionen auf.FazitDie zementfreie K-TEP ermöglicht eine nachhaltige Wiederaufnahme sportlicher Aktivitäten ohne erhöhtes Lockerungsrisiko im 5-Jahres-Verlauf. Die Ergebnisse sind hilfreich für die patientennahe Aufklärung über realistische Erwartungen nach der Operation.

**Fazit:**

Die Implantation einer zementfreien K-TEP ermöglicht eine nachhaltige Wiederaufnahme sportlicher Aktivitäten mit einer signifikanten Reduktion der Beschwerden. Im 5‑jährigen Verlauf traten keine Lockerungen auf. Die Ergebnisse dieser Studie können in der Patientenberatung genutzt werden, um realistische Erwartungen an die postoperative sportliche Leistungsfähigkeit zu vermitteln.

**Graphic abstract:**

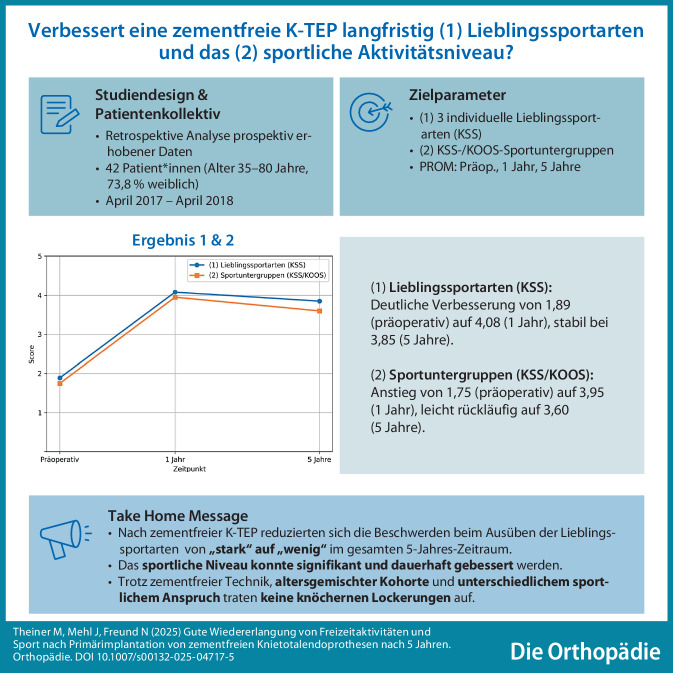

**Zusatzmaterial online:**

Die Online-Version dieses Beitrags (10.1007/s00132-025-04717-5) enthält umfangreiches Zusatzmaterial.

## Abstract (en)

**Introduction:**

The ability to resume sporting activities after total knee arthroplasty (TKA) is a key concern for many patients. While cementless implantation techniques are gaining relevance, long-term data on their impact on physical activity are limited. This study aimed to evaluate individual leisure activities and functional outcomes following cementless TKA over a five-year period.

**Materials and methods:**

A retrospective analysis of prospectively collected data was conducted on 42 patients (age 35–80 years) who underwent primary cementless TKA (Attune, DePuy) between April 2017 and April 2018. For each patient, the three most relevant sports were identified using the Knee Society Score (KSS) and compared pre- and postoperatively (1 and 5 years). Additionally, activity-related subscales of the KSS and KOOS (Knee injury and Osteoarthritis Outcome Score) were evaluated. Statistical analyses included Wilcoxon, Friedman, and Spearman correlation tests.

**Results:**

Patient-reported outcome measures (PROMs) showed significant improvements in sports-related performance (*p* 0.05) at 1 and 5 years postoperatively. Pain during favorite sports decreased from “severe” to “mild.” KSS activity subgroups improved from “severe–moderate” to “moderate–mild,” and KOOS subgroups from “extreme–severe” to “moderate.” KSS and KOOS scores showed strong correlation. No aseptic loosening or periprosthetic infections were observed.ConclusionCementless TKA allows for a sustained return to sporting activity with significant symptom reduction over a five-year period, without increased risk of implant loosening. These findings support realistic patient counseling regarding postoperative expectations.

**Discussion:**

This study demonstrates that patients benefit long term from a significant improvement in athletic ability after cementless TKA. Recent studies suggest that cementless techniques are not only equivalent to cemented approaches but may even be superior in certain aspects. The low loosening rate further supports the use of cementless TKA in physically active patients.

**Conclusion:**

Cementless TKA allows for a sustained return to athletic activities with a significant reduction in discomfort. No loosening occurred over the 5‑year period. The results of this study can aid in patient counseling to set realistic expectations regarding postoperative athletic performance.

## Einleitung

Eine zentrale Fragestellung im Rahmen der Planung einer Knietotalendoprothese (K-TEP) betrifft die postoperative Wiederaufnahme sportlicher Aktivitäten. Steigende Implantationszahlen sowie ein wachsender Anspruch an körperliche Aktivität im Alltag und in der Freizeit erhöhen die Relevanz dieses Aspekts [1, 2]. Ein Wiedererlangen der Alltags- sowie Freizeitaktivität nach immobilisierender Kniegelenksarthrose bringt nicht nur Lebensqualität wieder, sondern erhöht auch die Lebenserwartung signifikant [3]. Zahlreiche Studien belegen eine signifikante Schmerzreduktion und Funktionsverbesserung nach K-TEP. Die vorliegende Arbeit untersucht jedoch im Speziellen die sportlichen Parameter vor und nach Prothesenimplantation, um dadurch eine Quantifizierung der sportlichen Prognose für die Patienten und damit auch eine Entscheidungshilfe für die Operation bieten zu können.

Während zur zementierten Technik bereits vergleichbare Untersuchungen vorliegen [4, 5], bestehen für die zementfreie Implantation weiterhin Vorbehalte, insbesondere hinsichtlich eines potenziell erhöhten Lockerungsrisikos [6].

Aktuelle Erkenntnisse deuten jedoch darauf hin, dass insbesondere jüngere und aktivere Patienten funktionell von einer zementfreien Verankerung profitieren könnten [7]. Ebenso zeigen aktuelle Studien keine erhöhte Revisionsrate bei sportlich aktiven Patienten mit zementfreier K‑TEP [8].

Das primäre Ziel der vorliegenden Arbeit ist die Evaluation der Wiederaufnahme individueller sportlicher Freizeitaktivitäten über einen Zeitraum von 5 Jahren nach zementfreier K‑TEP. Als sekundärer Endpunkt wurde die Änderung der sportlichen und physikalischen Leistungsfähigkeit anhand validierter PROM über 5 Jahre nach zementfreier K-TEP-Implantation untersucht.

## Material und Methoden

Es wurde eine retrospektive Studie von prospektiv gesammeltem Patientendaten, welche eine zementfreie Knieprothesenimplantation (Attune, Fa. DePuy, Raynham, MA, USA) erhielten, durchgeführt. Anzumerken ist, dass die Datenerhebung für eine andere Studie in Zusammenarbeit mit der Firma DePuy in unserem Haus durchgeführt wurde. Die vorliegende Studie nutzte diese gesammelten Daten und es wurden die sportlichen Subgruppen der PROM des KOOS- und KSS-Scores ausgewertet. Dabei wurde ein Beobachtungszeitraum über 5 Jahre gewählt und die Studie war monozentrisch.

Einschlusskriterien wurden definiert: 35–80 Jahre, nichtinflammatorische Arthrose mit Untersucherindikation, freiwilliges Einverständnis, nicht bettlägerig, Patient versteht die Studie und kann diese durchführen, kann lesen und verstehen.

Ausschlusskriterien: Schwangerschaft, kontralaterale Seite bereits in Studie, kontralaterale Amputation, vorangegangene Schlittenprothese, Patellaoberflächenersatz oder kniegelenksnahe Osteotomie ipsilateral, radikuläre Schmerzen ipsilateral, andere Studienteilnahme in den letzten 3 Monaten, Frühpensionierungsbegehren, Drogen‑/Alkoholabhängigkeit in den letzten 5 Jahren oder psychische Vorerkrankung, muskelrelaxierende Medikamente bei Krankheiten wie Fibromyalgie oder Polymyalgie, schwere neurologische und muskuloskelettale Erkrankung mit Gang- und Belastungsstörung (MS, muskuläre Dystrophie, Charcot), entzündliche Arthritis wie Psoriasis und systemischer Lupus, weniger als 5 Jahre Lebenserwartung aufgrund von schweren Komorbiditäten, unkontrollierte Gicht.

Insgesamt erfüllten 47 Patienten, welche zwischen April 2017 und April 2018 eine primäre und zementfreie K-TEP erhielten, die Einschlusskriterien und wurden in die Studie eingeschlossen. Das „loss to follow-up“ betrug *n* = 0. Es zeigten sich 2 „drop outs“ (1 kleinzelliges Lungenkarzinom, 1 periprothetische Fraktur nach 4 Jahren). Bei 2 Patienten kam es im Verlauf zu einer sekundären ligamentären Insuffizienz, die eine operative Revision erforderlich machte. Diese Fälle wurden als Komplikation mit Revisionsindikation dokumentiert. Die präoperativen PROM eines Studienteilnehmers sind verloren gegangen, weshalb auch dieser nicht in der Studie berücksichtigt werden konnte. Ein Studienteilnehmer entwickelte im Verlauf eine Arthrofibrose und wurde arthroskopisch revidiert, konnte jedoch in der Studie beibehalten werden.

Endgültig wurden somit Daten von 42 Patienten in der vorliegenden Studie ausgewertet.

Das Eingriffshaus ist ein zertifiziertes Endoprothetikzentrum der Maximalversorgung. Alle Operationen wurden von EndoCert-Hauptoperateuren durchgeführt. Alle Patienten erhielten nach circa 3 Monaten einen Reha-Aufenthalt zur Optimierung der Rehabilitation. Eine passive Gelenkmotorschiene erhielten die Patienten solange die Flexion unter 60° betrug, stationär sowie auch zuhause.

### „Patient reported outcomes“

Für die erste Fragestellung wurden aus dem KSS-Score die Variablen der „Möglichen Aktivitäten“ entnommen. Bei dieser Subgruppe wählten die Patienten 3 aus 17 möglichen Freizeitaktivitäten und Sportarten aus, die für sie im Alltag und Leben am wichtigsten waren und bewerteten den Beschwerdegrad bei der aktuellen Durchführung davon. Dadurch entstehen pro Befragungstermin 3 Werte, von welchem der Mittelwert gebildet wurde. Dabei hat diese ordinale Skalierung 6 Variablen; welche von „kniebedingt nicht möglich“ (= 0) zu „gar keine Einschränkung“ (= 5) reicht.

Für die sekundäre Fragestellung wurden die sportlichen Untergruppen von 2 unterschiedlichen Fragebögen ausgewertet. Einerseits wurden aus dem KSS-Score alle mit Sport assoziierten Untergruppen „Grundaktivitäten“, „spezielle Aktivitäten“ und die zuvor auch verwendeten „möglichen Aktivitäten“ untersucht, was insgesamt 14 Werte pro Befragungstermin ergab. Erneut wurde ein Mittelwert aus diesen Variablen gebildet. Es handelt sich um denselben Score wie oben beschrieben, weshalb die ordinalen Variablen analog dazu sind. Zudem wurden aus dem KOOS-Score alle mit Sport assoziierten Untergruppen „Aktivitäten des täglichen Lebens“ und „Funktionalität bei Sport und Freizeitaktivitäten“ ausgewertet, welche insgesamt 22 Werte pro Befragungstermin ergaben. Für die spätere Auswertung wurde auch hier der Mittelwert gebildet. Dabei hat diese ordinale Skalierung 5 Variablen, welche von „keine Beschwerden“ (= 0) zu „immer“ (= 4) reichen. Die Werte der beiden Fragebögen können daher nicht direkt miteinander verglichen werden, da sie eine unterschiedliche Variablenanzahl haben und reziprok sind.

Die Patienten füllten in der Originalerhebung die kompletten KSS- und KOOS-Scores präoperativ, sowie nach 6 Wochen, 3 Monaten, 1 Jahr, 3 Jahren und 5 Jahren postoperativ aus.

Für die vorliegende Studie wurden die folgenden 3 Zeitpunkte evaluiert: präoperativ, 1. Jahr und 5. Jahr postoperativ. Diese Zeitpunkte waren für die Untersuchung unserer Fragestellungen relevant.

### Statistik

Zunächst konnten alle 3 Gruppen (KSS-Mögliche Aktivitäten, KSS-Sportuntergruppen, KOOS-Sportuntergruppen) mit dem Kolmogorov–Smirnov-Wahrscheinlichkeitsverteilungstest als normalverteilt berechnet werden. Der Vergleich der Variablen einer Gruppe an 2 Zeitpunkten (präoperativ und 5. Jahr postoperativ) wurde mit dem Wilcoxon-Test durchgeführt. Für den Vergleich der statistischen Signifikanz zwischen allen 3 Zeitpunkten eines Probanden (präoperativ, 1. Jahr und 5. Jahr postoperativ) wurde der Friedmann-Test durchgeführt . Es wurde eine zusätzliche Untergruppenbestimmung zwischen 2 Altersgruppen mit dem Scheidewert 65 Jahre zum Zeitpunkt der Implantation bestimmt.

Für die vereinfachte Lesbarkeit wurde in den Tabellen und Boxplots die „KSS-Mögliche Aktivitäten“ in „KSS Akt.“ unbenannt. Die „KSS- und KOOS-Sportuntergruppen“ wurden als jeweils „KSS“ und „KOOS“ umbenannt, obwohl sie nur die sportlichen Abschnitte der Fragebögen beinhalten. Zusätzlich erfolgte eine Korrelationstestung zwischen den Ergebnissen der beiden PROM der KSS- und KOOS-Sportuntergruppen mit dem Spearmen-Test.

Die Statistikberechnungen wurden mit dem kostenfreien Programm „Python“ (Python Software Foundation, Delaware, USA) durchgeführt. Für die errechneten Werte in dieser Arbeit wurde bis auf die 2. Kommastelle gerundet. Als statistisch signifikanter Unterschied in den vergleichenden Tests wurde ein *p*-Wert von unter 0,05 definiert. Eine Übersicht der Ergebnisse sind der Übersichtstabelle online unter Supplementary Information zu entnehmen. 

## Ergebnisse

Von den 42 Patienten waren 31 (73,8 %) Frauen und 11 (26,2 %) Männer. Wie oben beschrieben wurden 2 Altersgruppen definiert (Tab. 1).

**Tab. 1:** Demographische Daten der Studienpopulation

Variable	Ergebnis
Anzahl	42
Geschlecht	F = 31 (73,8 %); M = 11 (26,2 %)
Alter + STD	64,02 ± 9,34 (min. 35, max. 79)
Unter 65 Jahre	25 (59,52 %)
Über 65 Jahre	17 (40,48 %)

Beim Wilcoxon-Test zeigten sich eine statistisch signifikante Verbesserung von präoperativ zum 5‑Jahres-Zeitpunkt postoperativ innerhalb der 3 Gruppen (und auch Altersgruppen).

Beim Friedman-Test zeigten sich alle Vergleiche der Variablen zwischen präoperativ, 1. Jahr und 5. Jahr postoperativ innerhalb der 3 Gruppen (und Altersgruppen) statistisch signifikant (Tab. 2–4).

**Tab. 2:** Übersichtstabelle KSS (Knee Society Score) Aktiv mit statistischer Signifikanzbewertung

	Präoperativ	1 Jahr	5 Jahre	Sig. 5 Jahre	Sig. Werte
KSS Aktiv (gesamt)	1,89 ± 0,79	4,08 ± 0,62	3,85 ± 1,10	*p* < 0,00001	*p* < 0,00001
KSS Aktiv (< 65 Jahre)	1,84 ± 0,56	3,73 ± 0,96	3,69 ± 1,00	*p* = 00003	*p* < 0,00001
KSS Aktiv (> 65 Jahre)	1,88 ± 0,83	3,72 ± 0,80	3,58 ± 0,89	*p* < 0,00001	*p* < 0,00001

-Sig. 5 Jahre: *p*-Wert zwischen präoperativ und 5 Jahr postoperativ; Sig. Werte: *p*-Wert, welcher statistische Signifikanz zwischen allen 3 Werten angibt

### KSS aktiv.

**Abb. 1:**
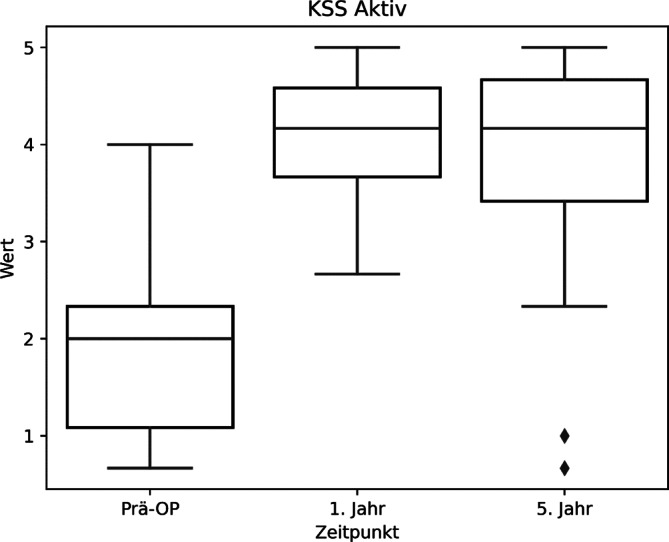
KSS (Knee Society Score) Aktiv, „Mögliche Aktivitäten“ Werte Boxplot

Dabei ist die ordinalen Skalierung im KSS wie folgt: 0 = nicht möglich, 1 = sehr starke Beschwerden, 2 = starke Beschwerden, 3 = mittelmäßige Beschwerden, 4 = wenig Beschwerden, 5 = keine Beschwerden. Analog zur Wertetabelle können wir also entnehmen, dass sich durch die Operation die präferierten Sportarten der Patienten von „starke Beschwerden“ zu „wenig Beschwerden“ gebessert haben (Abb. 1; Tab. 2). Damit kann die Alternativhypothese für die erste Fragestellung bestätigt werden.

**Abb. 2:**
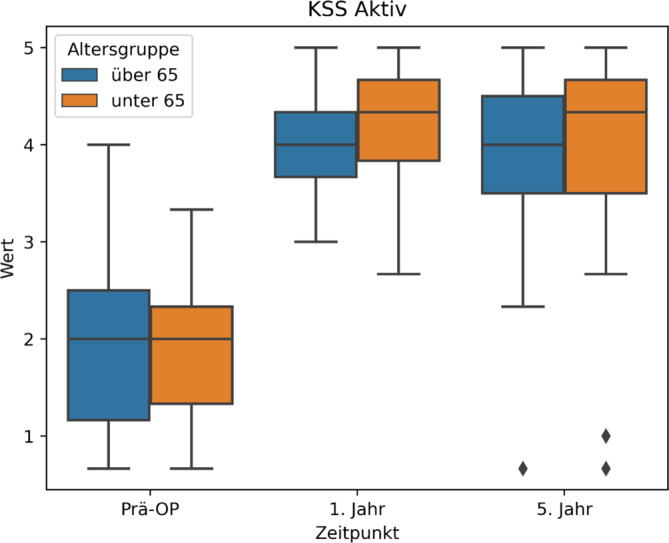
KSS (Knee Society Score) Aktiv Boxplot, Altersgruppen < > 65 Jahre

Es zeigen sich auch im Vergleich zwischen den beiden Altersgruppen ähnliche Ergebnisse (Tab. 2; Abb. 2).

**Tab. 3:** Übersichtstabelle KSS (Knee Society Score) mit statistischer Signifikanzbewertung

	Präoperativ	1 Jahr	5 Jahre	Sig. 5 Jahre	Sig. Werte
KSS (gesamt)	1,86 ± 0,68	3,73 ± 0,88	3,64 ± 0,94	*p* < 0,00001	*p* < 0,00001
KSS (< 65 Jahre)	1,87 ± 0,70	4,12 ± 0,65	3,88 ± 1,15	*p* < 0,00001	*p* < 0,00001
KSS (> 65 Jahre)	1,92 ± 0,91	4,04 ± 0,61	3,81 ± 1,08	*p* < 0,00003	*p* < 0,00002

-Sig. 5 Jahre: *p*-Wert zwischen präoperativ und 5 Jahr postoperativ; Sig. Werte: *p*-Wert, welcher statistische Signifikanz zwischen allen 3 Werten angibt

**Abb. 3:**
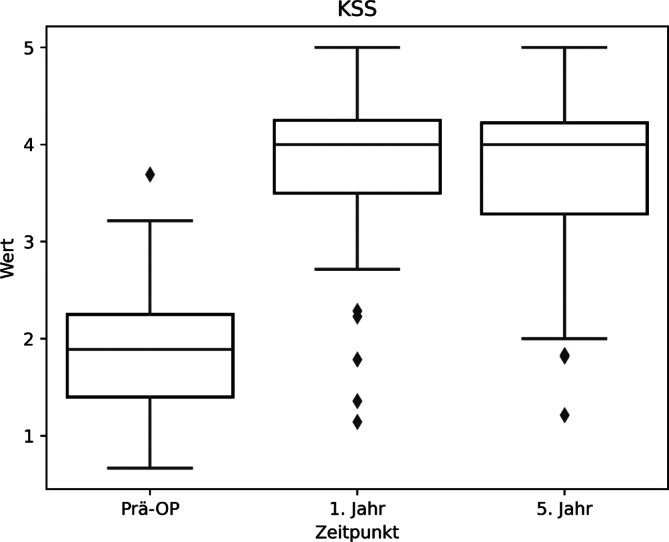
KSS(Knee Society Score)-Werte Boxplot

### KSS sport.

Analog zur Wertetabelle können wir also entnehmen, dass sich durch die Operation das sportliche Niveau der Patienten laut dem KSS-Score von „sehr starke bis starke Beschwerden“ zu „wenig bis mittelmäßige Beschwerden“ gebessert hat (Abb. 3; Tab. 3).

**Abb. 4:**
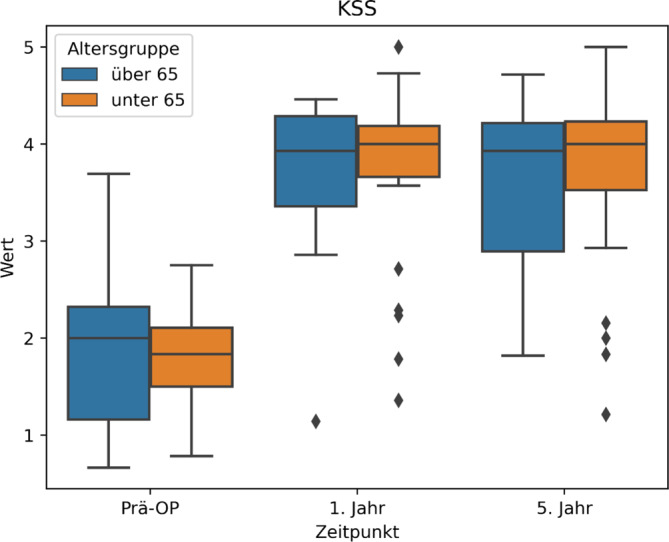
KSS(Knee Society Score)-Boxplot, Altersgruppen < > 65 Jahre

Es zeigen sich auch im Vergleich zwischen den beiden Altersgruppen sehr ähnliche Ergebnisse (Tab. 3; Abb. 4).

**Tab. 4:** Übersichtstabelle KOOS (Knee Injury und Osteoarthritis Outcome Score) mit statistischer Signifikanzbewertung

	Präoperativ	1 Jahr	5 Jahre	Sig. 5 Jahre	Sig. Werte
KOOS (gesamt)	3,58 ± 0,61	1,87 ± 0,68	1,86 ± 0,78	*p* < 0,00001	*p* < 0,00001
KOOS (< 65 Jahre)	3,59 ± 0,57	1,88 ± 0,66	1,80 ± 0,76	*p* < 0,00001	*p* < 0,00001
KOOS (> 65 Jahre)	3,55 ± 0,68	1,85 ± 0,73	1,94 ± 0,83	*p* < 0,00003	*p* < 0,00002

-Sig. 5 Jahre: *p*-Wert zwischen präoperativ und 5 Jahr postoperativ; Sig. Werte: *p*-Wert, welcher statistische Signifikanz zwischen allen 3 Werten angibt

**Abb. 5:**
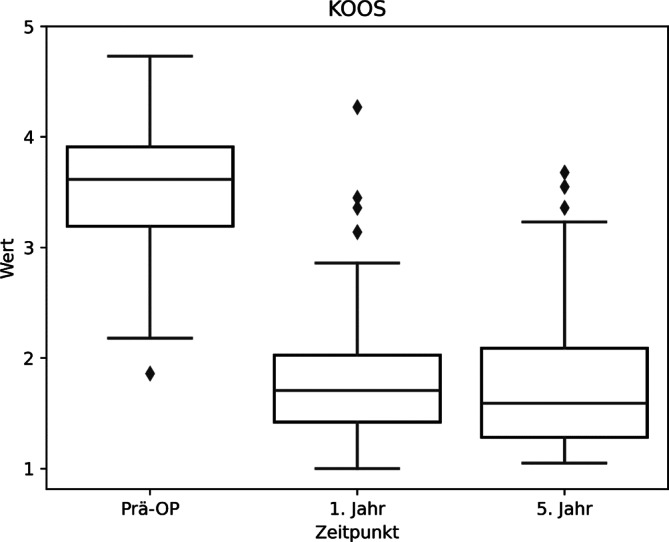
KOOS(Knee Injury und Osteoarthritis Outcome Score)-Boxplot

### KOOS sport.

Dabei ist die ordinale Skalierung im KOOS wie folgt: 4 = extreme Beschwerden, 3 = schwere Beschwerden, 2 = moderate Beschwerden, 1 = milde Beschwerden, 0 = keine Beschwerden.

Analog zur Wertetabelle können wir also entnehmen, dass durch die Operation sich das sportliche Niveau der Patienten laut dem KOOS-Score von „extreme bis starke Beschwerden“ zu „moderate Beschwerden“ gebessert hat (Abb. 5; Tab. 4).

**Abb. 6:**
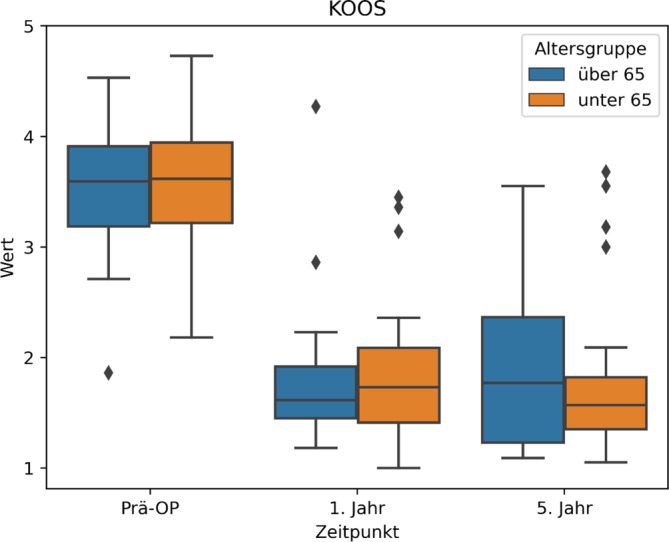
KOOS(Knee Injury und Osteoarthritis Outcome Score)-Boxplot, Altersgruppen < > 65 Jahre

Es zeigen sich auch im Vergleich zwischen den beiden verschiedenen Altersgruppen ähnliche Ergebnisse (Abb. 6). Betrachtet man alle 3 Boxplots, werden die Mittelwerte in allen 3 Gruppen durch wenige Ausreiser insgesamt leicht verschlechtert. Tendenziell zeigt die „jüngeren“ Altersgruppe nach 5 Jahren leicht gebesserte Beschwerden in allen 3 Gruppen.

Mit dem Spearman-Korrelationskoeffizient-Test wird die Korrelation zwischen den Werten der „sportlichen Aktivitäten“ von KSS und KOOS berechnet (siehe Grafik online unter Supplementary Information). Die negativen Korrelationswerte in der Tab. 5 erschließen sich aus dem reziproken Bewertungssystem der beiden PROM.

**Tab. 5:** Spearman-Korrelationskoeffizient KSS(Knee Society Score)/KOOS(Knee Injury und Osteoarthritis Outcome Score)-Wertetabelle

KSS/KOOS	Präoperativ	1. Jahr	5. Jahr
Korrelationskoeffizient	−0,61	−0,84	−0,88
*p*-Wert	< 0,00001	< 0,00001	< 0,00001

Mit −0,61 präoperativ zeigt sich lediglich, dass die beiden PROM beim „beschwerdereichen“ Zeitpunkt vor der Operation sich etwas unterscheiden. Es ist jedoch trotzdem noch eine gute Vergleichbarkeit der Werte präoperativ bei stärkeren Beschwerden gegeben.

Es zeigt sich mit einem Korrelationskoeffizient von −0,84 und −0,88 postoperativ eine sehr hohe Homogenität der Ergebnisse der beiden PROM.

Kombiniert man nun die sehr guten Ergebnisse des KSS- und KOOS-„Sportliche Aktivitäten“ mit der Erkenntnis der ausgezeichneten Korrelation postoperativ kann die Alternativhypothese auch für die 2. Fragestellung bestätigt werden.

Während des gesamten Beobachtungsverlaufs zeigten sich keine periprothetischen Infekte oder aseptischen Lockerungen. 2 Patienten erhielten eine Revision aufgrund ligamentärer Insuffizienz auf ein Prothesensystem mit höherem Kopplungsgrad.

Die 3 bevorzugten Aktivitäten/Sportarten der Teilnehmer waren Spazierengehen (*n* = 33), Gartenarbeit (*n* = 27) und Radfahren (*n* = 20).

## Diskussion

Die wichtigste Erkenntnis aus dieser Arbeit ist, dass die Teilnehmer ihre 3 Lieblingsaktivitäten bzw. -sportarten postoperativ deutlich besser ausüben können und auch die insgesamte sportlichen Fähigkeit sich signifikant besserte.

Trotz der zementfreien Technik zeigten sich während des Beobachtungszeitraums keine aseptischen Lockerungen. Die 3 Revisionen aufgrund der ligamentären Insuffizienz (*n* = 2) und der periprothetischen Fraktur (*n* = 1) wurden nicht als Versagen der zementfreien Implantationstechnik gewertet, da sie unabhängig von der knöchernen Integration auftraten.

In allen 3 Gruppen haben sich interessanterweise die Werte nach 5 Jahren im Vergleich zu 1 Jahr postoperativ minimal verschlechtert. Initial glaubten wir, dass die insgesamte Verschlechterung des Allgemeinzustandes bei den älteren Patienten Grund für dieses Phänomen sein könnte.

Jedoch zeigt sich dieser Trend auch in der Altersgruppe unter 65 Jahre. Auch war die Altersgruppe über 65 Jahre im KOOS die einzige Untergruppierung, in welcher sich sogar eine leichte Besserung zeigte.

In einer Vergleichsarbeit mit zementierter Technik zeigte sich interessanterweise lediglich bei den unter 60-Jährigen eine leichte Verschlechterung des Aktivitätsniveaus im 5‑Jahres-Verlauf [5]. Natürlich ist eine erneute Sportausübung nach der K-TEP auch abhängig vom präoperativen Allgemeinzustand und den Nebenerkrankungen des Bewegungssystems [9]. In der ersten Fragestellung ging es daher auch darum, ob der bereits somatisch ältere Patient wieder seine Freizeitaktivität, wie im Garten arbeiten, ausüben kann.

In den meisten Lehrbüchern gilt die zementierte Technik in der K-TEP als Goldstandard und wird von älteren Metaanalysen auch dahingehend bestätigt [10].

Rezente Metaanalysen jedoch zeigen nicht nur eine Ebenbürtigkeit, sondern auch bessere Langzeitrehabilitation nach zementfreier Technik [11]. Eine rezente, große Metaanalyse aus Kanada von Aaron G. Chen zeigt zudem eine signifikant geringere Revisionsrate [12].

In unserem Haus wird aufgrund der zunehmenden Studienlage größtenteils zementfrei operiert.

Wenn sich der Trend fortführt, wird wahrscheinlich in naher Zukunft die Frage nach der Altersgrenze für zementfreie Knieprothesen laut. Zu diskutieren gilt, ob eventuell diese Grenze analog zur Hüfttotalendoprothetik oft mit 75 Jahre gesetzt werden soll (abhängig von Osteoporose, Geschlecht etc.). Dabei ist sicherlich die differenzierte Kinematik und das unterschiedliche Belastungsprofil der beiden Gelenke zu bedenken, die bisher eigentlich die Gründe für die zementaffine Vorgehensweise in der K-TEP waren. Eine rezente Arbeit, welche die zementfreie Technik bei fortgeschrittenem Alter untersuchte, konnte im kurzfristigen Nachverfolgungszeitraum von 2 Jahren keine schlechteren Ergebnisse erkennen [13].

Aufgrund des aktuell zunehmenden Trends zur zementfreien K-TEP-Implantation sind die Resultate dieser Arbeit daher umso interessanter [10, 13].

Obwohl die Ergebnisse sehr gut sind, zeigte sich bei uns kein komplett beschwerdefreies Patientenkollektiv postoperativ. Diese Ergebnisse sind kongruent zu bereits vorliegenden anderen zementierten wie zementfreien Studien [5, 8]. Ein wichtiger Aspekt ist daher eine klare Kommunikation mit den Patienten, einerseits mit Erfragung der Erwartung des Patienten nach K-TEP, andererseits realistischer Darlegung der postoperativen Situation hinsichtlich Beschwerdeverbesserung.

Auch bei noch sportlich aktiven Patienten mit ansonsten starken Arthrosebeschwerden kann die K-TEP empfohlen werden, da das sportliche Niveau postoperativ angehoben werden kann [14].

Unsere Empfehlung postoperativ gilt lediglich für „Low-impact“-Sportarten. Die Ausführung von „High-impact“-Sportarten kann nämlich zur Halbierung der Standzeit aufgrund von aseptischer Lockerung führen [2]. Hier muss erwähnt werden, dass auch Sportarten wie Squash oder Tennis auswählbar waren, welche zu den „High-impact“-Sportarten gehören.

Stärken dieser Studie sind, dass ein relativ langer Beobachtungszeitraum von 5 Jahren gewählt wurde. Da die Datenerhebung der PROM primär für eine andere Studie mit anderer Fragestellung stattgefunden hat, kann ein möglicher Interviewer-Bias ausgeschlossen werden. Die Verwendung von zwei verschiedene PROM mit postoperativ ausgezeichneter Korrelation stärkt das Ergebnis.

Eine Altersdifferenzierung wurde ebenfalls durchgeführt. Auch ist die spezifische Fragestellung mit klarem Outcome für die Nutzbarkeit der Ergebnisse in der Sprechstunde essenziell.

Mit den Ergebnissen dieser Studie kann dem Patienten reproduzierbar der starke Benefit einer K-TEP für das sportliche Aktivitätsniveau bestätigt werden. Die Lieblingsaktivitäten des Patienten können wieder aufgenommen und auch nach 5 Jahren Beobachtungszeitraum ausgeführt werden. Laut unseren Ergebnissen zeigen sich bei zementfreier Operationstechnik auch bei sportlicher Aktivität sehr gute Ergebnisse und keine Lockerungen im mittelfristigen Zeitraum.

### Schwächen

Die größte Schwäche stellt das Fehlen einer Kontrollgruppe dar. Auch ist es keine prospektive Studie im engeren Sinn gewesen, zudem wäre eine größere Fallzahl von Vorteil gewesen.

## Fazit für die Praxis

- Nach zementfreier Knietotalendoprothese besserten sich die Beschwerden der Patienten von „stark“ zu „wenig“ beim Ausüben ihrer Lieblingssportarten im gesamten Beobachtungsraum.
- Es zeigt sich ein dauerhaftes statistisch signifikantes Anheben des sportlichen Niveaus.
- Trotz zementfreier Technik, gemischtem Alter und sportlichem Anspruch zeigten sich im 5‑Jahres-Verlauf dieser Arbeit keine knöchernen Lockerungen.

## Supplementary Information

Spearman-Korrelationskoeffizient KSS/KOOS-Grafik

Übersichtstabelle: Statistische Signifikanzbewertung aller 3 Gruppen mit Altersgruppen

## Data Availability

Die im Rahmen dieser Studie erhobenen Daten enthalten sensible personenbezogene Informationen und können daher nicht öffentlich zugänglich gemacht werden. Eine Weitergabe ist nur in anonymisierter Form und nach Prüfung eines begründeten Antrags möglich. Die Daten sind in einem Repositorium mit Zugangskontrolle gespeichert und können bei berechtigtem Interesse über den korrespondierenden Autor angefragt werden. Die Einwilligung der Studienteilnehmer zur Datennutzung und -weitergabe wurde vor Beginn der Datenerhebung gemäß den ethischen Richtlinien eingeholt.

## Abkürzungen

KOOS: Knee Injury and Osteoarthritis Outcome Score
KSS: Knee Society Score
K-TEP: Knie-Totalendoprothese
PROM: Patient-Reported Outcome Measure
TKA: Total Knee Arthroplasty
