# Supplementary figures and images for: Good recovery of leisure activities and sport after primary implantation of cementless knee arthroplasty after 5 years: A retrospective study
Source: Orthopadie (Heidelb). 2025 Sep 17;55(3):221–9. [Article in German] doi: 10.1007/s00132-025-04717-5 (PMC12946324; doi:10.1007/s00132-025-04717-5)

**Tabelle 16:** Spearman Korrelations-Koeffizient KSS / KOOS Grafik

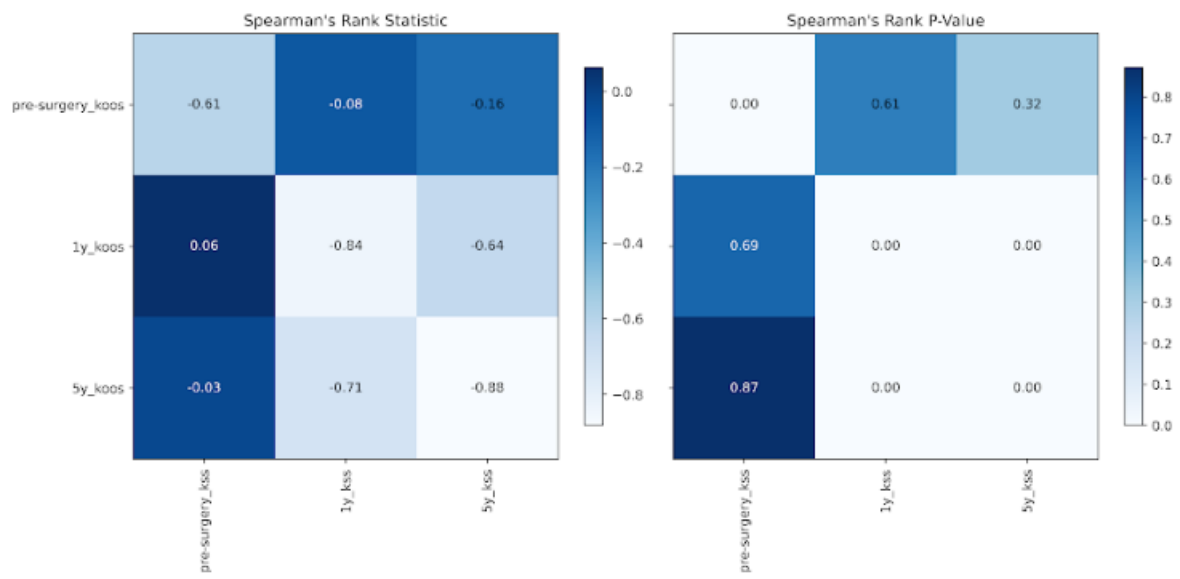

Supplement: Supplementary file 1 — Spearman-Korrelationskoeffizient KSS/KOOS-Grafik [file 132_2025_4717_MOESM1_ESM.pdf]
