# Supplementary material for: Good recovery of leisure activities and sport after primary implantation of cementless knee arthroplasty after 5 years: A retrospective study
Source: Orthopadie (Heidelb). 2025 Sep 17;55(3):221–9. [Article in German] doi: 10.1007/s00132-025-04717-5 (PMC12946324; doi:10.1007/s00132-025-04717-5)
Supplement: Supplementary file 2 — Übersichtstabelle: Statistische Signifikanzbewertung aller 3 Gruppen mit Altersgruppen [file 132_2025_4717_MOESM2_ESM.pdf]

Übersichtstabelle: Statistische Signifikanzbewertung aller 3 Gruppen mit Altersgruppen

Kategorie	Test	Berechnung	Wert
KSS Aktiv	Friedman	stat.	52,35
		p-Wert	< 0,00001
	prä-OP< Wilcoxon 5. Jahr	stat.	806,5
		p-Wert	0,00001
KSS Aktiv, unter 65 Jahre	Friedman	stat.	29,26436782
		p-Wert	< 0,00001
	prä-OP< Wilcoxon 5. Jahr	Stat.	250
		p-Wert	0,00003
KSS Aktiv, über 65 Jahre	Friedman	Stat.	23,20547945
		p-Wert	< 0,00001
	prä-OP< Wilcoxon 5. Jahr	stat.	166,5
		p-Wert	0,00021
KSS	Friedman	stat.	48,08187135
		p-Wert	< 0,00001
	prä-OP< Wilcoxon 5. Jahr	stat.	919
		p-Wert	< 0,00001
KSS, unter 65 Jahre	Friedman	stat.	30,58947368
		p-Wert	< 0,00001
	prä-OP< Wilcoxon 5. Jahr	stat.	299
		p-Wert	< 0,00001
KSS, über 65 Jahre	Friedman	stat.	17,78947368
		p-Wert	0,00014
	prä-OP< Wilcoxon 5. Jahr	stat.	178
		p-Wert	0,00013
KOOS	Friedman	stat.	58,07185629
		p-Wert	< 0,00001
	prä-OP< Wilcoxon 5. Jahr	stat.	899
		p-Wert	< 0,00001
KOOS, unter 65 Jahre	Friedman	stat.	37,41052632

		p-Wert	< 0,00001
	prä-OP< Wilcoxon 5. Jahr	stat.	300
		p-Wert	< 0,00001
KOOS, über 65 Jahre	Friedman	stat.	21,33333333
		p-Wert	0,00002
	Wilcoxon 5. Jahr>prä-OP	stat.	167
		p-Wert	0,00003
